# Patients’ views on changes in doctor-patient communication between 1982 and 2001: a mixed-methods study

**DOI:** 10.1186/1471-2296-13-80

**Published:** 2012-08-08

**Authors:** Ligaya Butalid, Peter F M Verhaak, Hennie R Boeije, Jozien M Bensing

**Affiliations:** 1 NIVEL, Netherlands Institute for Health Services Research, PO Box 1568, 3500, BN, Utrecht, The Netherlands; 2 Department of General Practice, Faculty of Medical Sciences, University of Groningen, Groningen, The Netherlands; 3 Department of Methodology and Statistics, Faculty of Social and Behavioural Sciences, Utrecht University, Utrecht, The Netherlands; 4 Department of Psychology, Faculty of Social and Behavioural Sciences, Utrecht University, Utrecht, The Netherlands

**Keywords:** Quality of care, Doctor-patient communication, Analogue patients, General practice, Video observation, Mixed-methods design

## Abstract

**Background:**

Doctor-patient communication has been influenced over time by factors such as the rise of evidence-based medicine and a growing emphasis on patient-centred care. Despite disputes in the literature on the tension between evidence-based medicine and patient-centered medicine, patients’ views on what constitutes high quality of doctor-patient communication are seldom an explicit topic for research. The aim of this study is to examine whether analogue patients (lay people judging videotaped consultations) perceive shifts in the quality of doctor-patient communication over a twenty-year period.

**Methods:**

Analogue patients (N = 108) assessed 189 videotaped general practice consultations from two periods (1982–1984 and 2000–2001). They provided ratings on three dimensions (scale 1–10) and gave written feedback. With a mixed-methods research design, we examined these assessments quantitatively (in relation to observed communication coded with RIAS) and qualitatively.

**Results:**

1) The quantitative analyses showed that biomedical communication and rapport building were positively associated with the quality assessments of videotaped consultations from the first period, but not from the second. Psychosocial communication and personal remarks were related to positive quality assessments of both periods; 2) the qualitative analyses showed that in both periods, participants provided the same balance between positive and negative comments. Listening, giving support, and showing respect were considered equally important in both periods. We identified shifts in the participants’ observations on how GPs explained things to the patient, the division of roles and responsibilities, and the emphasis on problem-focused communication (first period) versus solution-focused communication (last period).

**Conclusion:**

Analogue patients recognize shifts in the quality of doctor-patient communication from two different periods, including a shift from problem-focused communication to solution-focused communication, and they value an egalitarian doctor-patient relationship. The two research methods were complementary; based on the quantitative analyses we found shifts in communication, which we confirmed and specified in our qualitative analyses.

## Background

The way general practitioners (GPs) in the Netherlands communicate with their patients has been subject to trends and changes [1-3]. One of the important changes is the growing emphasis on evidence-based medicine. In 1989, the Dutch College of General Practitioners published the first national clinical guidelines [4-6]. Today, there are one hundred different clinical guidelines for general practitioners [7]. In addition, other developments in society at large and health care in particular, such as changes in morbidity (more chronic diseases), power balances (more egalitarian relationships), and accessibility of medical information (via the Internet) may have influenced how doctors and patients interact in medical consultations [8]. Topics such as shared decision-making and the development of evidence-based tools that support the involvement of patients in health care decisions have gained the interest of GPs and other health care providers [9]. Despite these developments in patient-centred care, it was found that doctor-patient communication in hypertension consultations has become more task-oriented in recent decades [1]. Patients talked less, while GPs provided more biomedical information and exhibit fewer concerns and worries in more recent consultations. In addition, GPs and patients perceived an improvement over time in the quality of doctor-patient communication [3]. The ideological agreement on the relevance of more patient-centred health care seems not to be automatically translated in more egalitarian relationships within the medical consultation room. Therefore, the importance of finding a balance between evidence-based medicine and patient-centred care has been emphasized by researchers and health professionals [10-13].

The goal of medicine is to correctly address health problems perceived by patients [14]. Although there does not seem to be any discussion on this central position of patients in health care, problems remain on finding proper quality assessments methods for patients [15,16]. However, studies show that lay people are competent to assess quality of care, that their assessments have an added value over ratings given exclusively by professionals or researchers, and that they are able to express their opinions about health care issues [17-22]. The term ‘analogue patients’ is used in communication studies to define lay people who rate video-taped medical consultations (real or scripted) while taking on the patient role [23-26]. Analogue patients’ perceptions of communication were found to generally overlap with clinical patients’ perceptions, which imply that analogue patients can be used as proxies for assessing doctor-patient communication [26]. Moreover, it has also been shown that lay people or patients have other priorities as compared to the physicians by whom they are treated [27]. These studies suggest that patients’ views can and should be fully utilised when studying which communicative aspects contribute to the quality of doctor-patient communication. In this study, we therefore focus on the patient’s perspective, which is based on experiential knowledge and may reveal different priorities and preferences compared to professionals [27-29].

Quality of doctor-patient communication is a multidimensional concept which involves biomedical and psychosocial aspects of medical care, but also involves facets of the interaction itself. Moreover, fostering the doctor-patient relationship is considered an essential and universal value within medical practice [30]. In addition, doctor-patient communication can be considered to be a combination of observable verbal and non-verbal behaviours and elements that are more difficult to observe or quantify [31]. Untrained patients may base their judgments on dimensions of interactions that are mostly intangible. In an attempt to grasp the observable as well as the more intangible aspects that contribute to the quality of doctor-patient communication, qualitative methods to examine patients’ views can be valuable and complementary to quantitative approaches [32-34].

The aim of this study is to examine whether analogue patients perceive shifts in the quality of doctor-patient communication and how these shifts may be defined. We investigated which communicative aspects of GP consultations were valued by analogue patients when rating the quality of communication from two periods: 1982–1984 versus 2000–2001.

## Methods

Analogue patients assessed the quality of doctor-patient communication during consultations from two periods. We focused on hypertension in general practice, since different dimensions of quality are clearly identifiable when dealing with hypertension; the quality of hypertension care depends on biomedical aspects of communication, but also on psychosocial dimensions [35]. The first batch consisted of consultations videotaped in 1982–1984. The second batch was videotaped in 2000–2001. To be able to clearly distinguish two periods, we selected consultations from two batches with an interval of almost 20 years. In the first period, clinical guidelines were not yet nationally implemented, while GPs in the second period were already very familiar with working with guidelines. This mixed-methods study consisted of two parts: 1) Participants rated the consultations quantitatively on three dimensions of quality of communication. Subsequent analysis examined whether the ratings of both periods were related to communicative behaviour as coded with the Roter Interaction Analysis System (RIAS); and 2) Participants provided negative and positive comments regarding the doctor-patient communication in the consultation, which were analysed by means of qualitative research methods. We used the software package MAXQDA2007 to conduct these qualitative analyses.

### Videotaped consultations

Based on the International Classification of Primary Care (ICPC), we selected videotaped consultations with hypertension patients (ICPC-codes K85-K87) from a larger dataset of two cohorts of random general practice consultations. We focused on hypertension consultations, because hypertension care involves both biomedical and psychosocial dimensions. The first cohort consisted of all hypertension consultations, selected from a random sample of 1,569 videotaped consultation in 1982–1984 (n = 103) [1,36-38]. However, due to technical deterioration of some videotaped consultations, only 81 consultations (recorded by 23 GPs) were useable for the quality assessments. The second dataset was recorded in 2000–2001 (n = 2,794) and consisted likewise of a random sample of general practice consultations [1,39]. From this dataset, we selected every first hypertension consultation from each of the 108 participating GPs (n = 108).

The patients in the selected consultations showed no differences in age and gender between the two study samples. The mean age was 58.5 (sd = 14.80) and 61.4 (sd = 14.66) years, respectively (n.s.) and 65% versus 63% of the sample was female (n.s.). In both samples the vast majority of the consultations were repeat visits. All physicians in the selected consultations were trained in general practice and the majority (92% versus 94%) had more than 5 years experience. In the first study sample (1982–1984), all of the physicians (N = 23) were male and in the second study sample (2000–2001), 80 were male and 28 were female (74% versus 26%). In the Netherlands, routine care for hypertension patients is delivered in general practice. The study was carried out in accordance with Dutch privacy legislation. All participating physicians and patients who were videotaped during their consultation gave their informed consent.

### Participants

Analogue patients with hypertension assessed videotaped consultations of both periods individually in the period from April 2010 to July 2010. People were recruited through advertisements on health related internet web pages as well as via flyers placed in health care settings (general practices, pharmacies). Participants who had previously been involved in other health research projects conducted by NIVEL were actively approached by mail. All participants met the following criteria: diagnosed with hypertension by a physician, consulted a general practitioner at least once in the past year, not involved in a health care related lawsuit or legal complaint procedure, and being able to understand and speak the Dutch language.

In total, 108 participants with hypertension (age 24–80; 73 female and 35 male) completed the quality assessments of the videotaped consultations. See Table 1 for background characteristics of the participants. Most participants (90%) did not have previous experience with health research and 15 participants (14%) were members of a patient organisation. All signed a statement of confidentiality in advance. Participants were instructed to signal when they recognized the doctor or patient on the video. In those cases the video would be stopped. However, this happened only once. Before starting the actual assessments of the videotaped consultations, the participants underwent a short training program in which the rating scale was explained and a typical consultation (not part of the sample) was shown to practice with the assessment scale.

**Table 1:** Background characteristics of the patient observers

**Background characteristics**	**Patient observers with hypertension (N = 108)**
Gender	
Female	73 (68 %)
Male	35 (32 %)
Age	
< 40	2 (2 %)
40 – 49	12 (11 %)
50 – 59	46 (43 %)
60 – 69	39 (36 %)
70 – 79	9 (8 %)
Education level	
Primary education	2 (2 %)
Secondary education	59 (54.5 %)
Third-level education	47 (43.5 %)
Employment	
Retired	35 (32 %)
Employed	31 (29 %)
Self-employed	5 (5 %)
Other (student, housewife, job seeker)	37 (34 %)
Native background	
Dutch	96 (89 %)
First generation migrant	6 (5.5 %)
Second generation migrant	6 (5.5 %)
Health	
Using medication for hypertension	81 (75 %)
Comorbidity other chronic disease	50 (46 %)
Health care use	
Contact with GP in last two months	76 (70 %)
Contact with medical specialist in past year	72 (67 %)

### Part I: Quantitative study

#### Quality assessments by participants

Each participant viewed 8–12 consultations (randomly assigned from both periods, but with a total duration of approximately 90 minutes) in order for each consultation in the sample to be rated 5 or 6 times. The total number of observations was 1,027. We asked participants to individually assess the consultations on three dimensions of quality of communication. A rating scale from 1 (very poor) to 10 (excellent) was used. The dimensions assessed by the participants were biomedical quality of communication, psychosocial quality of communication and quality of interpersonal behaviour. These three dimensions were previously also assessed by GP observers with a similar assessment protocol [3,37]. The assessments consisted of a question form with three separate questions: *“How do you judge the biomedical / psychosocial / interpersonal quality of this consultation?”*. For the assessments of the biomedical dimension, participants were instructed to consider the clarity of any medical explanations given by the GP. Second, the psychosocial dimension referred to the way non-somatic aspects related to the complaint were addressed, such as stress-related factors in the origin of hypertension and psychosocial problems caused by hypertension or its treatment. Third, the interpersonal quality referred to the way in which the GP succeeded in building an open and secure relationship with the patient. We noticed that patients did not have any difficulties recognizing these aspects of hypertension care and were therefore capable of distinguishing all three dimensions based on their experiential knowledge.

#### Communicative behaviour of the GP coded with RIAS

Doctor-patient communication had already been coded for another project using RIAS [1], and these data were available for secondary analyses. RIAS is a widely-used international observation system with proven validity and reliability [40]. In the RIAS-coding system the communication units are defined as utterances - the smallest discriminable speech segment to which a classification may be assigned [41,42]. The RIAS distinguishes task-oriented behaviour (asking questions, giving information, counselling) from affect-oriented (personal remarks, showing concern, rapport building) and process-oriented (giving directions, partnership building) behaviour. The categories in RIAS are mutually exclusive and classify all utterances during a medical interaction and are therefore suitable for analysing the composition of consultations in detail and examine the proportion of different communication categories within consultations. Although different observers coded the two samples of consultations, all coders had been extensively trained according to the same training protocol using the RIAS-manual [41,42]. The manual received an update between the two periods. However, there were no relevant changes between the manual of 1987 and 1993 [1]. To check on inter-observer reliability, approximately 10% of all videotaped consultations were coded by at least two observers. In both samples the inter-observer reliability of the RIAS categories was shown to be satisfactory to very good with Pearson’s r ranging from 0.72 to 0.99.

### Statistical analyses

To account for the multilevel structure of quality assessments nested within videotaped consultations and individual observers, multilevel regression analyses were applied. The effects of communicative behaviour of the GP during the consultations on the ratings by analogue patients were examined. These analyses were executed for both periods (1982–1984 and 2000–2001). It was also tested whether there were any effects of individual analogue patients’ characteristics (such as age, gender, perceived health). Since none of these effects were found to be significant, we decided to leave out these analyses in the result section of this paper.

### Part II: Qualitative study

#### Comments given by participants

We asked participants to individually provide for each consultation any negative and positive comments regarding the doctor-patient communication in the consultations. They were free to note anything that they considered relevant to the quality of the doctor-patient interaction. Textual analysis of written comments gave us the opportunity to examine independent opinions on different consultations. With this qualitative method, we were able to make a decent comparison between consultations from two periods. We noticed that the participants could easily relate to the hypertension consultations and generally did not have any difficulties in writing down their feedback. The median number of comments per participant was 19 (range of 1 to 43 comments), with only 9 participants writing down less than 10 comments. In total, the consultations from the first period received 627 positive comments (mean of 7.74 notes per consultation) and 433 negative comments (mean of 5.35 notes per consultation). The consultations from the second period received 772 positive comments (mean of 7.15 notes per consultation) and 443 negative comments (mean of 4.10 notes per consultation).

### Qualitative analyses

We performed the qualitative analysis in five steps: 1) construction of the code list; 2) coding the complete dataset of comments and examining the frequencies of the codes; 3) comparing the code frequencies between both periods; 4) identifying recurrent themes in the focus and terminology of comments; 5) comparing the identified themes between both periods.

*Step 1: construction of the code list.* Based on the comments of participants, a thematic analysis of quality aspects was conducted by two researchers (LB and HB), in order to construct a conceptual code list [43]. In a first round of open coding, the positive as well as the negative comments on ten consultations from both periods were coded by the two researchers independently. The three dimensions of quality of doctor-patient communication (biomedical, psychosocial and interpersonal) were used as a conceptual framework for the coding of the comments. However, the researchers were also free to identify any new dimensions of quality of doctor-patient communication while coding. For example, while the three dimensions of quality of communication were identified and further specified in different components, patients also mentioned general communication characteristics that were added as basic conditions for the three quality dimensions. Discussion of this first round of coding resulted in an initial list of codes, which was used and modified by the two researchers in a second round of coding. For example, one of the discussion points was whether or not to create a separate code ‘being involved’ in addition to the existing codes ‘offering continuity’, ‘treating respectfully’ and ‘reassuring’. In the final version we decided to add this code because involvement was indeed a different category as it appeared in the comments. The final code list is displayed in Table 2. 

**Table 2:** Final code list with definitions and examples per code

***General communication characteristics***		
**Code**	**Definition**	**Examples**
Preparing patient	Preparing and directing patients by announcing examination or provide structure in the consultation	*+ GP: “I am going to take your blood pressure”*
		*- Does not announce what he is going to examine*
Asking questions	Questions by the GP that refer to the medical complaint or psychosocial aspects related to the complaint	*+ The doctor asked about her leg cramps*
		*- Did not ask relevant questions*
Explaining	Giving explanations about the medical complaint, examination, or psychosocial aspects of the complaint	*+ Explains the function of the medicine*
		*- Did not mention the blood pressure after examination*
Working efficiently	Working efficiently and being organized	*+ Immediately comes to the point talking about the ECG*
		*- Was very busy with paper work before he could give attention to the patient*
Taking time for patient	Being patient and calm	*+ Takes a lot of time for the patient*
		*- Is fast, hurried, and uninterested*
Talking intelligibly	Any comments on talking intelligibly; patient unable to understand what GP is saying	*+ Clearly pronouncing the sentences because of patient’s deafness*
		*- Talking too softly and not finishing his sentences*
Communicating appropriately	General comments on communication and the words used by the GP	*+ Very relaxed communication between doctor and patient*
		*- GP is too nonchalant*
***Biomedical quality***		
**Code**	**Definition**	**Examples**
Decision making	Deciding on a treatment, giving advice, prescribing medicine	*+ Gives multiple options, lets the patient make a choice*
		*- Does not give an advice*
Performing correctly	Technically good performance, proceeding correctly	*+ Takes the initiative to measure blood pressure*
		*- Does not examine the shoulder*
***Psychosocial quality***		
**Code**	**Definition**	**Examples**
Being alert to psychosocial signals	Noticing psychosocial signals, paying attention to patient’s mental state	*+ He identifies the concerns of the patient*
		*- GP does not react when Mrs says that she does not sleep well because of tension*
Giving advice	Giving advice on psychosocial aspects of the complaint	*+ Patient gets a referral to psychologist*
		*- Only gives brief information about whether or not the patient can go back to work*
***Interpersonal quality***		
**Code**	**Definition**	**Examples**
Offering continuity	Being familiar with the patient and knowing patient’s personal background	*+ Recaps what was discussed in the past*
		*- Not well informed about the patient’s medical history*
Being involved	Showing sincere involvement and adopting a personal approach	*+ Asks how patient experienced her recent hospitalization*
		*- Very business-like*
Treating respectfully	Being polite; being friendly; taking time to greet patient	*+ Speaks very respectfully to older lady*
		*- Does not greet the patient at the start of the consultation*
Listening attentively	Paying full attention to patient; listening; showing interest; not permitting distraction by telephone interruptions	*+ Shows interest in the patient*
		*- There is not much eye contact*
Reassuring	Verbally and non-verbally showing reassurance and support	*+ Reassures patient by saying ‘You don’t have to worry’*
		*- Tense atmosphere; which does not reassure the patient*
Treating patient as equal	Taking patient seriously; not being arrogant or patronizing	*+ Takes the patient seriously*
		*- The GP talked about the patient and did not put much effort in establishing contact with the patient*
Following the patient’s story	Being patient-centered; reacting to the patient’s input; taking patient’s view into account	*+ Reacts to patient’s comments*
		*- Rejects all suggestions by the patient (e.g. taking vitamin supplements)*
Showing appraisal	Giving compliments; show appraisal	*+ Gives a compliment about quitting smoking*
		*- Did not react to the fact that patient lost weight*
Respecting privacy	Dealing correctly with confidentiality	*+ GP says: I’d rather give it [prescription] to the person who is going to use it*
		*- It is not professional to talk about other patients during the consultation*

*Step 2: examining frequencies of codes.* The constructed code list was used to code the total dataset of comments. LB performed the coding of the dataset, while HB randomly cross-checked assigned coding categories. We calculated the frequencies of comments assigned per code.

*Step 3: comparing frequencies of codes.* We identified the top 3 frequencies of each group of comments (positive versus negative comments and first versus second period) and we examined whether there were shifts in these top frequencies between the two periods.

*Step 4: identifying themes in comments.* We studied the content of the comments for each period separately, to identify overall themes. Themes were first identified by the two authors LB and HB, and then discussed with all authors.

*Step 5: comparing themes in comments.* Finally, we explored whether any changes had occurred in the focus and terminology of the identified themes in step 4 between the two periods.

## Results

### Part I: Quantitative study

#### Communicative determinants of quantitative assessments

For the first period, a positive relationship was found between the biomedical quality assessments by analogue patients and the number of biomedical questions and amount of information and counselling given by the GP during the consultation (see Table 3). However, this relationship was not visible for the second period. For the second period, none of the communication variables were found to be significantly associated with biomedical quality according to the assessments.

**Table 3:** Analogue patients’ assessments (biomedical, psychosocial and interpersonal quality) related to GPs’ communication (coded with RIAS)

	**Period 1982–1984 (N observations = 485)**						**Period 2000–2001 (N observations = 542)**					
	**Biomedical quality**		**Psychosocial quality**		**Interpersonal quality**		**Biomedical quality**		**Psychosocial quality**		**Interpersonal quality**	
**GP Communication**		*| Z|*		*| Z|*		*| Z|*		*| Z|*		*| Z|*		*| Z|*
Constant	6.07		5.36		5.93		6.80		5.77		6.46	
*Task-oriented communication*												
*(questions, information, counselling)*												
- Biomedical	**0.013**	2.14 *	0.008	0.95	0.007	0.88	0.004	0.98	−0.005	1.03	−0.003	0.64
- Psychosocial	0.018	1.79	**0.031**	2.30 *	**0.024**	**1.92**	0.004	0.36	**0.051**	3.71 ***	**0.026**	2.05 *
- Lifestyle	−0.019	1.46	0.006	0.31	−0.012	0.73	0.023	1.41	0.022	1.19	0.028	1.62
*Affect-oriented communication*												
- Personal remarks	0.014	1.37	0.024	1.73	**0.031**	2.43 *	−0.015	1.64	0.015	1.38	**0.020**	2.09 *
- Sharing concern	0.021	0.98	0.022	0.79	0.037	1.41	−0.025	0.53	0.005	0.09	−0.049	0.97
- Rapport building	0.002	0.82	**0.013**	3.31 **	**0.008**	2.18 *	0.003	0.72	**0.011**	**1.96**	0.008	1.55
- Disagreements	−0.047	0.90	−0.111	1.59	−0.112	1.73	0.082	0.49	−0.080	0.42	−0.065	0.37
*Process-oriented communication*												
- Giving directions	0.012	0.90	0.004	0.26	0.007	0.44	0.025	1.74	0.027	1.64	0.030	1.95
- Partnership building	−0.032	1.23	−0.040	1.14	−0.020	0.61	0.032	0.54	0.014	0.20	0.049	0.80

* p < .05; ** p < .01; *** p < .001.

Assessments of psychosocial quality were positively related to the number of psychosocial questions, and amount of information and counselling given by the GP in both periods. In addition, rapport building was positively associated with psychosocial quality in the first period (*B =* 0.013, *Z* = 3.31, *p* < .01). In the second period, the effect of rapport building was not significant, but showed a trend (*B* = 0.011, *Z* = 1.96, *p* = .05).

A positive relation between personal remarks by the GP and the interpersonal quality assessments was found in both periods. In addition, psychosocial communication was positively related to interpersonal quality assessments. Although the effect of psychosocial communication was only significant in the second period (*B* = 0.026, *Z* = 2.05, *p* < .05), psychosocial communication also showed a trend in the first period (*B* = 0.024, *Z* = 1.92, *p* = .06). Finally, rapport building was positively associated with interpersonal quality in the first period, but not in the second period.

To identify which part of the variance was located on the video level and observer level respectively, we calculated the intraclass correlations (ICC) on these levels for both periods. In the first period, the intraclass correlations on video level were 12% (biomedical), 23% (psychosocial) and 22% (interpersonal). The intraclass correlations on observer level were 26% (biomedical), 23% (psychosocial) and 19% (interpersonal). In the second period, the intraclass correlations on video level were 15% (biomedical), 16% (psychosocial), and 6% (interpersonal). The intraclass correlations on observer level were 20% (biomedical), 20% (psychosocial), and 17% (interpersonal). The variance on video level decreased for the psychosocial and interpersonal quality assessments, indicating more uniformity between consultations on these dimensions in the second period. The variance on observer level decreased for all three dimensions, indicating more agreement between observers on how to define quality of communication in recent consultations.

### Part II: Qualitative study

#### Examining and comparing frequencies of codes (step 2 and 3)

The frequencies and percentages of positive and negative comments for both periods are displayed in Table 4. The top 3 of codes most often given to positive comments were: asking questions, explaining clearly and performing correctly for the first period (1982–1984). The top 3 of codes given to positive comments shifted only slightly for the second period (2000–2001); codes most often given to positive comments were: asking questions, explaining clearly and reassuring. The top 3 codes given to negative comments remained the same between the two periods: explaining clearly, performing correctly and listening attentively. Especially the codes explaining clearly and performing correctly were given to positive as well as negative comments. Based on these top frequencies of assigned codes per group, we could not identify relevant shifts in the number of comments between the two periods; similar topics were positively and negatively mentioned when participants rated videotaped consultations from the two periods.

**Table 4:** Frequencies and percentages of positive and negative comments for both periods

	**Period 1982-1984**		**Period 2000-2001**	
	**+ Positive comments**	**- Negative comments**	**+ Positive comments**	**- Negative comments**
***General communication aspects***				
- Preparing patient	11 (2 %)	4 (1 %)	7 (1 %)	6 (1 %)
- Asking questions	**102 (16 %) ▴**	25 (6 %)	**132 (17 %) ▴**	27 (6 %)
- Explaining clearly	**90 (14 %) ▴**	**57 (13 %) ▼**	**141 (18 %) ▴**	**48 (11 %) ▼**
- Acting efficiently	6 (1 %)	23 (5 %)	17 (2 %)	26 (6 %)
- Taking time for patient	31 (5 %)	8 (2 %)	33 (4 %)	5 (1 %)
- Talking intelligibly	3 (1 %)	5 (1 %)	3 (0.5 %)	4 (1 %)
- Communicating appropriately	14 (2 %)	31 (7 %)	29 (4 %)	18 (4 %)
***Biomedical quality***				
- Making decisions	19 (3 %)	27 (6 %)	41 (5 %)	23 (5 %)
- Performing correctly	**67 (11 %) ▴**	**39 (9 %) ▼**	52 (7 %)	**48 (11 %) ▼**
***Psychosocial quality***				
- Being alert to psychosocial signals	22 (4 %)	13 (3 %)	20 (3 %)	16 (4 %)
- Giving advice	16 (3 %)	7 (2 %)	6 (1 %)	1 (0.5 %)
***Interpersonal quality***				
- Offering continuity	27 (4 %)	21 (5 %)	25 (3 %)	16 (4 %)
- Being involved	36 (6 %)	6 (1 %)	39 (5 %)	18 (4 %)
- Treating respectfully	27 (4 %)	30 (7 %)	15 (2 %)	18 (4 %)
- Listening attentively	47 (7 %)	**48 (11 %) ▼**	47 (6 %)	**54 (12 %) ▼**
- Reassuring	38 (6 %)	23 (5 %)	**66 (9 %) ▴**	41 (9 %)
- Treating patient as equal	26 (4 %)	33 (8 %)	17 (2 %)	22 (5 %)
- Following the patient’s story	37 (6 %)	25 (6 %)	64 (8 %)	46 (10 %)
- Showing appraisal	8 (1 %)	1 (1 %)	14 (2 %)	1 (0.5 %)
- Respecting privacy	0 (0 %)	6 (1 %)	1 (0.5 %)	5 (1 %)
**Total**	**627**	**433**	**772**	**443**

▴ Top 1–3 codes of positive comments.

▼ Top 1–3 codes of negative comments.

#### Themes of comments on consultations from 1982–1984 (step 4)

Our next step in the qualitative analysis was to examine the content of the comments for each period and to indicate overall themes. Analysis of the comments on the videotaped consultations of the first period identified seven overall themes that were visible across the different quality dimensions: clarity of explanations, asking for consent, responsibility of GP, problem-focused approach, active listening, supporting patient, and showing respect.

As instructed, many participants reacted on the clarity of explanations given by GPs. They criticized consultations in which explanations were either unclear or were completely lacking *“Does not give any explanation to the patient about complaint and cause”.* Second, we identified asking for consent in the decision-making process as a theme. Participants judged asking for patient’s agreement positively *“Asks whether the lady wants to take the medicine”.* Third, participants valued GPs’ initiatives to assume responsibility *“Takes the initiative to check the blood pressure”.* We also identified a problem-focused approach as a theme that emerged from the comments. Participants focused on whether GPs asked questions to get a complete picture of the problem *“Asks about any possible causes of the complaints”*, gave patients enough room to share their problem *“The doctor let the patient talk before taking her blood pressure”*, and followed the patient’s story. In addition, active listening *“Listens carefully to the complaint of dizziness”* and supporting the patient *“Indicates that patient most probably does not have anaemia, but agrees on doing a blood test to reassure the patient”* were themes mentioned in the comments. Last, showing respect was a theme that emerged *“Opens the door for patient, asks ‘how are you?’ and shakes hands to greet”*.

#### Themes of comments on consultations from 2000–2001 (step 4)

Analysis of the comments on the videotaped consultations of the second period identified seven overall themes that were visible across the different quality dimensions: clarity of explanations and giving reasons for advice, exploring patients’ preferences, shared responsibility, solution-focused approach, active listening, supporting patient, and showing respect.

Again, participants judged consultations on the clarity of explanations given by GPs, but in addition they also gave feedback on whether these explanations were accompanied by clear reasons *“Gives reasons for her advice”.* Second, we identified exploring patients’ preferences in the decision-making process as a point of focus *“GP asks: What do you want?”*. Consultations in which GPs provided alternative options also received positive remarks by patient observers*.* Third, there were comments regarding shared responsibility in the consultations from the second period *“GP says: Great that you are thinking about how to maintain your health”* and *“GP says: If you experience any problems with the medication, please come back sooner”.* However, even though participants consider sharing responsibility positively, GPs are also expected to be more proactive in monitoring the patient’s health *“There is no clear follow-up appointment”*. Furthermore, we identified a solution-focused approach in the second period. Participants looked at whether GPs actively work towards a solution for the health problem *“GP immediately takes action regarding the blood pressure”* and whether they work efficiently *“GP summarizes the complaints and takes the lead to prevent the patient from repeating her complaints over and over again”.* In addition, active listening and supporting the patient were themes visible in comments. Last, participants mentioned whether GPs showed respect towards patients *“GP says at start of consultation: Mr V., what can I do for you?”*.

#### Comparison between the two periods based on qualitative assessments (step 5)

**Figure 1:**
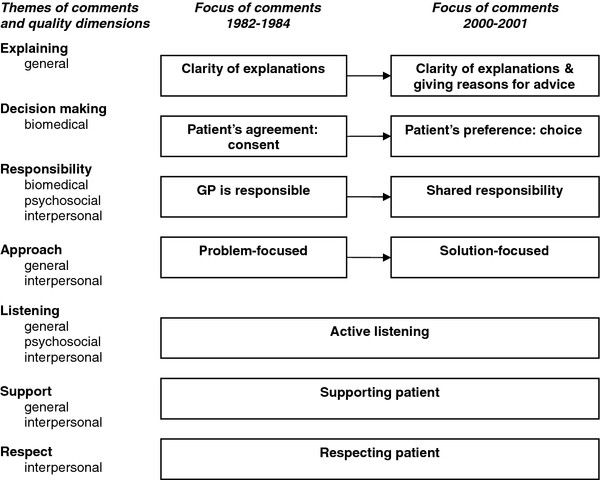
Comparison between the two periods: shifts and stabilities in focus and terminology of comments.

When comparing the themes identified from the comments on the two periods, we found that listening, giving support and showing respect were consistent themes in terms of focus in the quality assessments of analogue patients, as well as the terminology used. However, we recognized shifts in the participants’ observations on how GPs explained things to the patient, the decision-making process, the division of roles and responsibilities, and the emphasis on problem-focused communication in the first period versus solution-focused communication in the last period (see Figure 1).

Comparison between both periods on the theme explaining indicated a shift in focus in the analogue patients’ assessments. While comments on consultations from the first period emphasized clarity of explanations, comments regarding the second period emphasized clarity of explanations and giving reasons for the advice.

Furthermore, there was a shift in how participants assessed decision-making. In the first period, they focused on consent and commented on whether GPs asked for patients’ agreement to proposed treatments. In contrast, participants focused on choice in the second period and commented on whether patients’ preferences were taken into account and whether alternative treatment options were presented by GPs. This shift was mostly visible in the positive comments given by participants. Negative comments in both periods referred to the lack of checking for agreement or the absence of making a decision at all.

In addition, the theme of responsibility showed a shift in focus and terminology. Where GPs were considered to have the main responsibility in clinical decisions and monitoring patients’ health in the 1980s, we identified a focus on shared responsibility in the second period. This shift was mainly visible in the positive comments on both periods. The negative comments referred mostly to behaviour indicating that GPs were not proactive enough and not assuming responsibility during the consultation. This was also true for the second period, where shared responsibility was the main focus. Participants gave negative comments when GPs placed all the responsibility on the patient.

Last, the way participants assessed the approach of GPs changed between the two periods. Participants valued a problem-focused approach in the first period, while a solution-focused approach was valued in the second period. The first period received mostly positive comments regarding problem-focused behaviour, while negative comments could refer to not identifying the problem but also to the absence of working on a solution to the problem. In the second period, positive comments referred mostly to solution-focused behaviour, while negative comments referred to not being solution-focused or efficient, but could also refer to a lack or absence of problem-focused behaviour *“He did not let the patient finish”* or being too business-like in their approach towards the patient.

## Discussion

The assessments by analogue patients with hypertension of the quality of doctor-patient communication during consultations indicated that shifts had taken place between the first period and the second. This was visible from the quantitative analyses where we found that biomedical communication and rapport building were positively associated with the assessments of the first period, but not with those of the second period. In addition, we found less variation between consultations on psychosocial and interpersonal quality and more agreement between observers on how to define quality of communication in recent consultations. In the qualitative analyses, we identified shifts in focus and approach; the most important shifts being the shift from problem-focused to solution-focused communication and the shift towards a more egalitarian doctor-patient relationship. The findings of both research methods can be considered complementary; based on our quantitative results we found shifts, but we could not specify which communicative behaviour of the GP was valued more highly in the second period as opposed to the first period. Based on our qualitative results we confirmed and specified the content of shifts and we identified themes that were assessed differently in the second period compared to the first.

### Egalitarian doctor-patient relationships

The analogue patients valued GPs’ encouragement of patient involvement (emphasizing patient’s choice, shared responsibility, giving reasons for advice) in consultations from the second period. These findings are in line with the increased attention to patient involvement in general practice [44]; previous research showed that patients want more information from their physicians and may want to actively participate in decision-making processes [45].

Furthermore, participants judged shared responsibility positively, but also reacted negatively when the GP did not assume enough responsibility or failed to make a decision. This may seem contradictory; however, it illustrates that concepts such as responsibility and decision making consist of different components. Our findings indicate that analogue patients may distinguish between processes of involvement and decision outcomes, in line with recent literature on shared decision making [46,47]. Future guidelines and decision making tools should therefore respond to patients’ needs to be involved in decision processes, as opposed to focusing merely on decision outcomes.

### Affective communication

There were also some stable quality aspects that participants mentioned in both periods. The quantitative analyses showed that psychosocial communication and personal remarks were positively related to quality assessments of both periods. In the qualitative analyses, we did not identify any shift in focus of comments on the themes listening, support and respect. Previous studies also found that patients are sensitive in perceiving whether they are respected by their physicians and appreciate the opportunity and time given to present their concerns [48,49]. Our findings indicate that the analogue patients valued these aspects regardless of the period of consultations being assessed. Listening to the patients’ story, showing respect and giving support seem to be universal quality indicators from the patient’s perspective. The same results were found in an international multicentre study (GULiVER), where analogue patients from four different countries also put most emphasis on physicians’ affective communication [22,50]. Attention to fostering the doctor-patient relationship has been found to be continuously valued by patients; our study indicated that factors such as respect and listening are conditionally for a ‘good consultation’. However, in postgraduate GP training, it was found that trainees scored higher on more traditional communication skills (e.g. history taking) as compared to affective communication skills such as dealing with emotions and exploration of expectations and feelings [51]. Based on our findings we argue that GPs should prioritize the doctor-patient relationship and put more emphasis in affective communication and attitudinal factors.

### Strengths and limitations of the study

We used a combination of quantitative and qualitative research methods. This approach gave us the opportunity to study patients’ views on the quality of doctor-patient communication from different perspectives and enabled a more comprehensive examination of patients’ views including dimensions that may be less obvious [15,43]. In line with our expectations, the results of both methods were complementary; the qualitative analyses of the comments given by analogue patients revealed themes and specified the content of shifts which we were not able to measure with our quantitative data. Furthermore, we examined medical interactions using videotaped real-life general practice consultations with hypertension patients from two distinct periods. Thus, the findings referred to actual behaviour as perceived by uninvolved observers. In addition, the videotaped participants were not aware of the fact that the analyses would focus on hypertension consultations. Video recording is a valid method to examine doctor-patient communication: the influence of the video recorder on participants’ behaviour is marginal [52,53].

A possible limitation of the study is that the assessments of participants were performed retrospectively. Analogue patients judged videotaped consultations that took place approximately 10 or 30 years ago respectively, but they were also influenced by current knowledge and experience. Therefore, the context in which their ratings were conducted is different from the historical context of the videotaped consultations. Since it can be argued that expectations of what is considered a ‘good’ consultation are also subject to change over time, we cannot automatically assume that quality assessments would have been identical if analogue patients had also rated the consultations at the time of recording. Furthermore, our study concerned the communicative behaviour of the GP and not of the patient. However, we expected that the assessments would mainly focus on the GPs’ behaviour, since the camera was directed at the GP during the videotaped consultations. In line with this expectation, the notes of the participants referred mostly to the behaviour of the GP. However, to investigate the role of patients during consultations, future research studies should focus more explicitly on patients’ contribution to quality of doctor-patient communication. Our study indicates that independent observers such as analogue patients should be instructed explicitly to also reflect on patients’ behaviour during consultations. Another possible weakness is that the majority of the observed consultations concerned hypertension repeat visits and the participants were hypertension patients. Therefore, we need to be cautious with the generalizability of our findings. Future studies should therefore focus on different consultation types, in order to make more general assertions regarding changes in doctor-patient communication. Our study indicated the importance of affective communication by GPs in hypertension consultations, which prompts for further investigation on changes in affective communication during other consultation types, such as consultations about psychosocial problems as opposed to somatic problems.

## Conclusion

Summarizing, this study indicates that analogue patients recognize shifts in the quality of doctor-patient communication from two different periods, including a shift from problem-focused communication to solution-focused communication, and that they value an egalitarian doctor-patient relationship. Evidence-based medicine and attention to the process of shared decision making are aspects of the medical interaction that are valued by patients, as long as GPs do not lose sight of conditional factors such as listening and respect.

## Competing interests

The authors declare that they have no competing interests.

## Authors’ contributions

LB coordinated the analogue patients’ assessments, formulated the study questions, discussed core ideas, analysed the quantitative and qualitative data, and wrote the paper. PV designed the original study, discussed core ideas, and edited the paper. HB analysed the qualitative data and edited the paper. JB designed the original study, discussed core ideas, and edited the paper. The study adheres to the RATS guidelines on qualitative research. All authors have read and approved the manuscript.

## Pre-publication history

The pre-publication history for this paper can be accessed here:

http://www.biomedcentral.com/1471-2296/13/80/prepub
